# Deep Learning of Histopathology Images at the Single Cell Level

**DOI:** 10.3389/frai.2021.754641

**Published:** 2021-09-10

**Authors:** Kyubum Lee, John H. Lockhart, Mengyu Xie, Ritu Chaudhary, Robbert J. C. Slebos, Elsa R. Flores, Christine H. Chung, Aik Choon Tan

**Affiliations:** ^1^ Department of Biostatistics and Bioinformatics, H. Lee Moffitt Cancer Center and Research Institute, Tampa, FL, United States; ^2^ Department of Molecular Oncology, H. Lee Moffitt Cancer Center and Research Institute, Tampa, FL, United States; ^3^ Department of Head and Neck-Endocrine Oncology, H. Lee Moffitt Cancer Center and Research Institute, Tampa, FL, United States; ^4^ Cancer Biology and Evolution Program, H. Lee Moffitt Cancer Center and Research Institute, Tampa, FL, United States; ^5^ Molecular Medicine Program, H. Lee Moffitt Cancer Center and Research Institute, Tampa, FL, United States

**Keywords:** histopathology image analysis, deep learning, image data labeling, cell type classification, tumor immune microenvironment, tumor heterogeneity

## Abstract

The tumor immune microenvironment (TIME) encompasses many heterogeneous cell types that engage in extensive crosstalk among the cancer, immune, and stromal components. The spatial organization of these different cell types in TIME could be used as biomarkers for predicting drug responses, prognosis and metastasis. Recently, deep learning approaches have been widely used for digital histopathology images for cancer diagnoses and prognoses. Furthermore, some recent approaches have attempted to integrate spatial and molecular omics data to better characterize the TIME. In this review we focus on machine learning-based digital histopathology image analysis methods for characterizing tumor ecosystem. In this review, we will consider three different scales of histopathological analyses that machine learning can operate within: whole slide image (WSI)-level, region of interest (ROI)-level, and cell-level. We will systematically review the various machine learning methods in these three scales with a focus on cell-level analysis. We will provide a perspective of workflow on generating cell-level training data sets using immunohistochemistry markers to “weakly-label” the cell types. We will describe some common steps in the workflow of preparing the data, as well as some limitations of this approach. Finally, we will discuss future opportunities of integrating molecular omics data with digital histopathology images for characterizing tumor ecosystem.

## Introduction

In clinical settings, histopathology images are a critical source of primary data for pathologists to perform cancer diagnostic. For some cancer types, clinicians may decide treatment strategies based on histopathology images coupled with molecular assay data. With the widespread adoption of digital slide scanners in both clinical and preclinical settings, it is becoming increasingly common to digitize histology slides into high-resolutions images. Digital pathology, which is the process of digitizing histopathology images, creates a new “treasure trove of image data” for machine learning (ML). Machine learning can be utilized for various image analysis tasks that are routinely performed during histological analyses including detection, segmentation, and classification. Some commercial image analysis software already incorporates machine learning algorithms to assist researchers and clinicians in quantifying and segmenting histopathological images. These tools have greatly reduced the laborious and tedious manual work in image analysis and can reduce inter-observer variability in reaching diagnostic consensus (Tizhoosh et al., 2021).

Machine Learning which focuses on methods to construct computer programs that learn from data with respect to some class of tasks and a performance measure, has been widely applied in several challenging problems in bioinformatics due to the algorithm’s ability to extract complex relationships from high-dimensional data. Conventional machine learning methods (e.g. random forest, support vector machines) were limited by their ability to extract features from raw data, and in many cases, a feature selection step is needed to reduce dimensionality of the data. In addition, efforts have been invested in careful feature engineering and domain knowledge to construct informative features to train the model. However, some engineered features are difficult to interpret biologically and have limited utility in biomedical applications.

In early 2000, several breakthroughs including new types of algorithms (e.g., deep learning), availability of large datasets (e.g., open access and large digitized images), and advancements in computing power (e.g., graphical processing units) have reenergized the machine learning developments and applications in real-world problems (LeCun et al., 2015). Deep Learning (DL) is a family of new machine learning models composed of multiple processing layers that learn representations of data with multiple levels of abstraction without feature engineering. The ability of deep learning to discover intricate structure in large data sets powered by a backpropagation algorithm allows the machine to change its internal parameters to compute a representation in each layer from the previous layer. The “deep” in deep learning representing the number of layers used in the model to deconvolute the feature representation of the raw data. These methods have dramatically improved the state-of-the-art in multiple domains ranging from speech and text recognition to object detections in biomedical applications (Esteva et al., 2017; Lee et al., 2018; McKinney et al., 2020; Nagpal et al., 2020; Liu et al., 2021).

The field of cancer pathology is proving to be a supremely suitable proving ground for the development of machine learning models, in no small part due to the construction of publicly available, curated whole slide image (WSI) datasets from initiatives like the Cancer Genome Atlas (TCGA), Clinical Proteomic Tumor Analysis Consortium (CPTAC), and the Cancer Image Archive (TCIA) (Clark et al., 2013; Prior et al., 2013). The datasets contained within these repositories often include other related data, such as clinical characteristics, patient outcomes, molecular analyses, and other imaging modalities, in addition to the WSIs. These data can be utilized as target features, such as predicted progression-free survival duration, or even integrated into the machine learning model for higher dimensional analysis. The numerous types of cancer collected by these repositories allow researchers to focus their applications as narrowly or broadly as they desire, from single subtypes (e.g., lung adenocarcinoma) to pan-cancer analyses.

However, the majority of machine learning applications in this field rely on supervised learning methods based on clinical parameters or pathologists’ annotations to generate training datasets. Within supervised learning approaches, there exists several distinct resolutions of annotation required to generate a high-quality training dataset depending on the scale of the analysis. The ultimate goal of machine learning applications for histopathology is to generate clinically beneficial output, but this may be achieved in a wide variety of ways. For example, both a model designed to flag regions of concern for a pathologist to review in detail and a tool that identifies cancer patients that are likely to respond to immunotherapies by classification of immune cell types are likely to improve clinical outcomes, but these two models will require very different training datasets. In this review, we will consider three different scales of histopathological analyses that machine learning can operate within: WSI-level, region of interest (ROI)-level, and cell-level.

Many reviews have been published in describing the methods and applications of deep learning in pathological image analysis [see (Janowczyk and Madabhushi 2016; Dimitriou et al., 2019; Serag et al., 2019; Roohi et al., 2020; van der Laak et al., 2021)], however, none of these publications discussed or reviewed on the topic of training datasets preparation for ML/DL, which is the most crucial step in developing a useful model in histopathological image analysis. In addition, it is becoming clear that the tumor immune microenvironment (TIME) plays crucial role in determining cancer progression, metastasis, and response to treatment. Therefore, it is important to detect and classify the different immune cell types of the TIME in histopathological images. However, it is impractical to manually curate and annotate these individual cell types for training the model. To address this knowledge gap, we will discuss the basics of applying machine learning models for histopathological analysis within a cancer pathology setting, review currently published models and applications in the three different scales of histopathology analyses, and provide a simplified framework for the development of a cell-type classifier using weakly labeled datasets generated from immunolabeled slides. We aim for this review to be an approachable introduction to histopathological applications of machine learning/deep learning for clinicians, biologists, and data scientists, thereby encouraging further development of this interdisciplinary field.

## Machine Learning-Based Histopathology Image Analysis and Data Generation Methods

### Histopathology Image Data Preparation for Training Machine Learning Models

**FIGURE 1:**
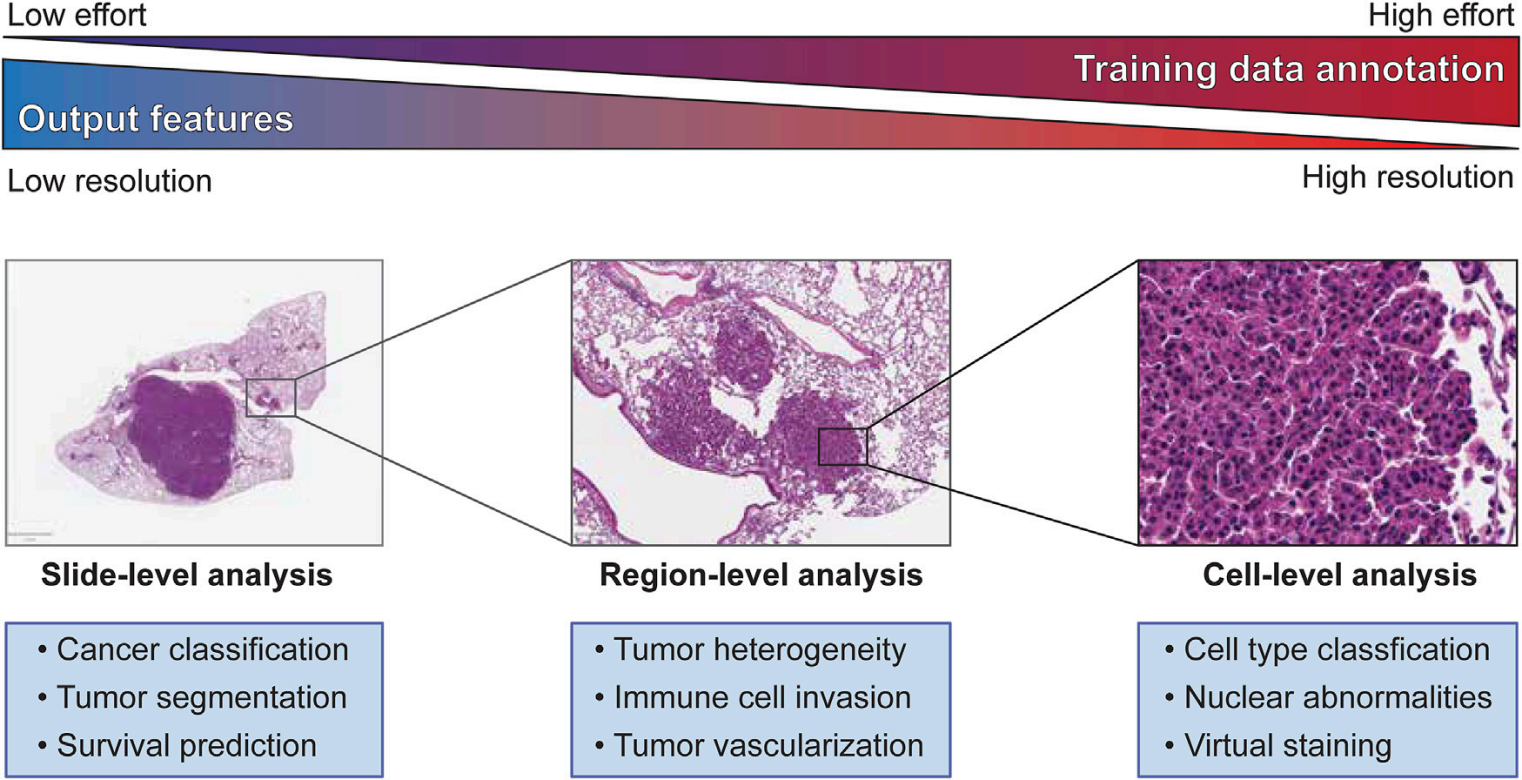
Slide-, Region-, and Cell-level analyses on tumor histopathology images and its characteristics.

**FIGURE 2:**
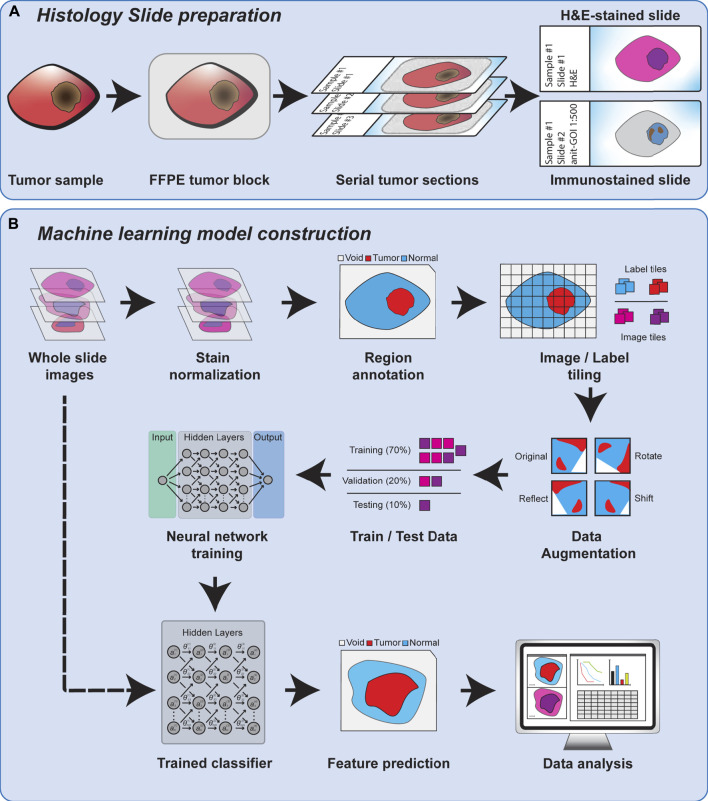
An overview of histology slide preparation and machine learning model construction process.

Regardless of the feature scale of the ML analysis, it is also important to understand how the input data is prepared. Histopathology analysis is most commonly performed using sections of tissue collected during biopsy or after surgical resection. These tissues are typically preserved by formalin fixation and paraffin embedding (FFPE) to preserve their morphology, and subsequently sliced into thin sections on glass slides for further processing. The tissue sections are commonly subjected to chemical staining to highlight specific tissue or cellular features, such as nuclei or proteins of interest. Normal hematoxylin staining produces intense purple staining the nuclei of cells while eosin is used to counterstain the remaining cytoplasm of cells a vivid pink (Figure 1). Hematoxylin and eosin (H&E) staining is employed in nearly every histological workup and is therefore the most common type of histological image used as inputs for machine learning models. Figure 2 shows the overview of the histopathology slide preparation and machine learning model construction.

Training a machine learning model for image segmentation requires a large amount of high-quality, labeled images as a training dataset. Therefore, building an effective training dataset requires a careful balancing of data quantity, data quality, and cost. In comparison to many other fields that utilize computer vision, the amount of publicly available histopathology data suitable for training a machine learning model is quite limited. As previously mentioned, there is a growing number of datasets from consortia like TCGA, but many published studies also rely on in-house datasets for training and testing their machine learning models. This scarcity of data is compounded by the need for trained experts capable of producing accurate annotations that capture the defining features of the model’s target classes. The difficulty of these annotations in terms of both the rater’s expertise and the effort required to create increases sharply between whole slide-level, region-level, and cell-level analyses (Figure 1). Constructing the best training dataset will require annotations at the same level as the target outputs of the machine learning model (i.e., cell-level analysis performs best with cell-level annotation), but approaches like crowd-sourcing annotations may work as an alternative to expert annotation by sacrificing annotation quality for quantity (Amgad et al., 2019). For some applications, lower resolution annotations, such as annotating a region as a single class for training a cell-level classifier or labeling of cell centroids, may also be used to generate “weakly-labeled” annotations for model training. Alternatively, histology slides can be immunolabeled to identify specific cells or features of interest that can then be used to weakly label an adjacent, registered H&E image, an approach we will discuss later in this review. While these challenges may incline researchers to annotate at the lowest usable resolution to expedite model training, it should be noted that many analyses require the abstraction of higher resolution classifications to a lower level to produce biologically meaningful results (e.g., calculating the density of a cell type within a region after classification of individual cells).

Despite the complexities of training dataset generation, the number of applications and tools that utilize machine learning models in cancer pathology has grown rapidly over the last few years. This pace seems likely to continue and perhaps even increase as more datasets are made publicly available through cancer consortia and computational challenges like BreastPathQ (Petrick et al., 2021) or CAMELYON (Litjens et al., 2018). In the following three sections, we will cover published examples of machine learning applications and discuss the strengths, limitations, and considerations of each analysis scale level. We also summarized the published examples in Table 1.

**TABLE 1:** Selected deep learning-based histopathology image analysis studies.

Publication	Input image type	Training annotations	Deep learning architecture	Prediction output	Other functions	Training dataset size	Level
Zhang et al. (2019)	Tiled images from whole slide (H&E)	Pathologist marking tumor and normal areas, input description text	CNN	Probability of being tumor at Pixel level	Text query of images using pathological terms	913 whole slides	WSI
			Attention module	Attention area			
			RNN	Text description			
TOAD Lu et al. (2021)	Whole slide	Tumor of origin	CNN	Primary or metastatic		22,833 whole slides from 18 cancer types +6,499 test slides	WSI
		Sex					
				Tumor of origin			
		Primary or metastatic	Attention module				
				Attention areas			
Kalra et al. (2020)	Whole slide (H&E, frozen section)	Primary diagnosis	DenseNet	Relevant images ranked by similarity	Images features extracted as barcodes	29,120 whole slides from 32 cancer types (TCGA)	WSI
HE2RNA Schmauch et al. (2020)	Whole slide images	RNA expression for training	multilayer perceptron	RNA expression	Spatial mapping of gene expression	8,725 samples from 28 cancer types	WSI
Saltz et al. (2018)	Whole slide images (H&E)	Pathologist marking regions of lymphocytes and necrosis	CNN	TIL maps (Computational Staining)		5,455 images from 13 cancer types (TCGA)	ROI
Le et al. (2020)	Whole slide images (H&E)	Pathologist marking tumor regions, TIL annotations from Saltz et al. study	34-layer ResNet, 16-layer VGG, and Inception v4	Tumor probability and TIL probability heatmaps		Cancer detection: 393 breast cancer images from SEER and TCGA	ROI
						Lymphocyte detection: 1090 invasive breast cancer from TCGA	
Lockhart et al. (2021)	Whole slide images (H&E)	Pathologist marking normal vs. tumor region (grade 1–4)	CNN(ResNet18)	Normal lung tissue/airways		In house images from mouse models	ROI
				Lung adenocarcinoma of different grades (1–4)			
ConvPath Wang et al. (2019)	Whole slide images (H&E)	Pathologist labeled ROI (tumor, stroma, lymphocytes)	CNN	“spatial map” of tumor cells, stromal cells and lymphocytes (limited to lung adenocarcinoma)		TCGA-LUAD(1337)	ROI/Cell level
						NLST(345)	
						Beijing(102)	
						SPORE(130)	
CRImage Failmezger et al. (2020)	Whole slide images (H&E)	Single cell annotations, OS, omics data	Topological tumor graphs (TTG), unsupervised deep learning framework (CNx)	Cell level classified and mapped slices for further analysis and hypothesis generating		400 SKCM (TCGA)	Cell level

### Image-Level Analysis Methods

One of the immediate applications of deep learning in cancer research is to generate a cancer diagnosis from WSI analysis. Zhang et al. (2019) developed an artificial intelligence system to effectively automate WSI analysis to assist pathologists in cancer diagnosis. The AI system consists of three main neural networks: the scanner network (s-net), the diagnose network (d-net) and the aggregator network (a-net). In brief, the s-net ingests the WSI as inputs and automatically detects tumor regions in the images. Convolutional neural networks (CNN) was used in the s-net to manage tumor detection and cellular-level characterization. Once the tumor regions were detected, it further segments into ROIs for diagnostics. The d-net takes the ROI from s-net as inputs, and further characterizes by extracting pathological features and showing feature-aware network attention to explain the network for interpretations. The d-net is developed by using fully connected recurrent neural networks (RNN). Finally, the a-net integrates all the characterized features and provides the final diagnosis. The a-net is implemented as a three-layer fully connected neural network that takes the features and predict the final labels. The authors also showed that their model could simultaneously generate pathological reports from the d-net. The authors trained this method using 913 H&E WSI slides obtained from TCGA Bladder Cancer and in-house slides. The authors showed that the prediction from the model matches the diagnoses from 17 pathologists. This study shows that machine learning/deep learning could be used to assist pathologists in cancer diagnostic from WSI.

Cancer of unknown primary (CUP) represents a group of cancers in which the primary anatomical site of tumor origin cannot be determined. Unsurprisingly, CUP poses challenges to determine the appropriate treatments and clinical care. To address this challenge, Lu et al. (2021) developed a deep learning-based algorithm known as Tumor Origin Assessment via Deep Learning (TOAD), with the goal to predict the tissue of origin of the primary tumor using routinely acquired histology images. Histology slides from patients were automatically segmented and divided into thousands of small image patches and fed into a convolutional neural network (CNN) with fixed pretrained parameters. The CNN serves as the encoder to extract a compact, descriptive feature vector from each image patch. TOAD uses an attention-based multiple-instance learning algorithm to learn to rank all of the tissue regions in the WSI using the feature vectors, and aggregates this information across the whole slide based on their relative importance, and assigning more weights to regions perceived to have high diagnostic value. The authors also included patient’s gender as an additional feature to further guide the classification of CUP. Based on this multi-branched network architecture and the multi-task learning objective, TOAD is able to predict both the tumor origin and distinguish primary from metastatic tumors. The authors trained TOAD on 22,833 WSIs spanning across 18 common origins of primary cancer, and tested TOAD on 6,499 WSIs with known primary tumor origins. TOAD achieved a top-1 accuracy of 0.83 and a top-3 accuracy of 0.96. Further testing TOAD on external test set of 682 samples showed that it achieved top-1 accuracy of 0.80 and a top-3 accuracy of 0.93. Finally, the authors tested TOAD on 317 cases of CUP, and found that their model predicted in concordance for 61% of cases and a top-3 agreement of 82%. The authors suggested that this model could be used to assist pathologists to perform CUP assignment, as well as other difficult cases of metastatic tumor assignment.

In another study, Kalra et al. (2020) developed a pan-cancer diagnostic consensus through searching histopathology images using machine learning (ML) approaches. The authors first indexed ∼30,000 WSI of 32 cancer types from TCGA using Yottixel, an image search engine previously developed by the authors (Kalra et al., 2020b). To index the WSI, the authors generated “bunch of barcodes” (BoB) index for each WSI instead of small patches of images. Because the dimensional reduction from patches of images to BoB, this indexing step accelerate the retrieval process and overcome the computation and storage of huge image files. The authors used DenseNet to extract the image patch and convert into a vector, and the BoB essentially is the binary form of the deep feature vector representations of each image patch. The authors illustrated the application of this machine learning approach on the TCGA WSI data, and showed that their method could retrieve relevant images with high accuracy (>90% on several cancer types). This study demonstrates that an alternative approach to query WSI in a database to retrieve relevant set of WSIs for potential cancer diagnosis, and particularly useful for rare cancer types.

### Region of Interest-Level Analysis Methods

In addition to classification and searching tasks at whole slide level, deep learning approaches are also able to provide insights at more granular level, or give more emphasis to particular regions of interest (ROI) that are most informative. ROIs may be defined as geographic regions (e.g., central, marginal areas), or areas that are biologically divergent (e.g., tumor vs. stromal area, areas of different tumor grades), or areas that are enriched for specific cell types such as lymphocytes. A variety of ML tools have been developed to identify and analyze these ROIs and can provide unique insights into the biological differences between ROIs.

Spatial patterns of tumor-infiltrating lymphocytes (TILs) have shown significant value to cancer diagnosis and prognosis, however manually recognizing of those patterns requires tremendous efforts. Aiming to reduce the manual efforts and scale-up analysis capacity, Saltz et al. (2018) constructed a pipeline that mapped TILs to 5,455 H&E stained images from 13 TCGA tumor types. Their pipeline comprises two CNN modules (a lymphocyte-infiltrated classification CNN-lymphocyte CNN-and a necrosis segmentation CNN), that were trained on pathologist-annotated images of lymphocytes and necrosis. The training process also involves pathologists’ feedback to improve performance. The CNNs combined outputs were used to produce TIL probability map that was then subjected to threshold adjustments to obtain the final TIL map. During testing, this pipeline achieved 0.95 area under the receiver operating characteristic curve (AUROC) which outperformed VGG16 network (0.92). Moreover, the authors compared the extracted TIL structure patterns with the molecular based estimation (i.e., CIBERSORT) and found it achieved ∼0.45 correlation coefficient in best performed cancer types (e.g., BLCA, SKCM) and ∼0.1–0.2 correlation coefficient in worst performed cancer types (e.g., UVM, PAAD).

Another group followed up the above-mentioned study and modified the deep learning architecture to especially focus on breast cancer cases. 198 high-resolution WSIs from the Surveillance, Epidemiology, and End Results (SEER) dataset and 195 annotated TCGA breast cancer WSIs were utilized for the cancer detection task, and 1,090 breast cancer WSIs annotated from Saltz study were used for TIL classification task. The authors adapted and compared three different architectures including 16-layer VGG (VGG16), the 34-layer ResNet (ResNet34), and the Inception-v4 network using accuracy, F1 score, and AUC as performance metrics. Overall, the ResNet34 was the best performer in both cancer detection task and lymphocyte detection task, even surpassing the Saltz study’s accuracy in the case of breast cancer. Using their ResNet34 model, Le et al. (2020) showed that their estimated TIL infiltration was a significant survival predictor.

In addition to assessing lymphocyte infiltration, users are also interested in evaluating tumor progression and heterogeneity based on the distribution of cancer cells of different grades in the whole slide. To this end, the Flores laboratory has developed a deep learning system-Grading of Lung Adenocarcinoma with Simultaneous Segmentation by an Artificial Intelligence (GLASS-AI) (Lockhart et al., 2021), based on preclinical lung adenocarcinoma models. A ResNet18-based CNN was trained to classify and map the normal lung tissue, normal airways, and the different grades (1–4) of lung adenocarcinoma in WSI of mouse lungs. The modal not only achieved a micro-F1 score of 0.81 on a pixel-by-pixel basis, but also uncovered a high degree of intratumor heterogeneity that was not reported by the pathologists. We are currently utilizing this pipeline in conjunction with spatial transcriptomic analysis and IHC to conduct mechanism investigations to reveal new therapeutic targets and prognostic markers.

### Cell-Level Analysis Methods

Understanding the spatial organization of different cell types in the tumor microenvironment (TME) provides information on cancer progression, metastasis, and response to treatment. Currently, this information could be provided by extensive immunolabeling of specific cell types or performing spatial transcriptomics, though this technology is still in its infancy. To compensate this, researchers have been actively developed innovative approaches to extract cell-level information from images.

To provide a deeper understanding about the spatial information of cells involved in stromal-immune interface, Failmezger et al. (2020) developed CRImage, a computational pathology pipeline used to classify cells in the H&E-stained specimens into stromal, immune or cancer cells. The authors performed the analysis on 400 melanoma specimens obtained from TCGA. The authors compared the estimated proportions of these cell types with independent measures of tumor purity, estimation of lymphocyte density by expert raters, computed immune cell types and pathway analyses. Using a set of independent single-cell annotations, the authors showed that the classifier to achieve 84.9% balanced accuracy (81.9% recall, 90.9% precision). By comparing the gene expression profiles of these samples, the authors demonstrated that samples with high lymphocyte percentage were enriched for immune-related pathways, validating the CRImage approach.

In another study, Wang et al. (2020) developed Histology-based Digital-Staining (HDS), a deep learning-based computation model, to segment the tumor, stroma, lymphocyte, macrophage, karyorrhexis, and red blood cell nuclei from standard H&E-stained pathology images. They applied HDS in lung adenocarcinoma H&E images to classify cell nuclei and extracted 48 cell spatial organization-related features that characterize the TME. Based on these features, they developed an accurate prognostic model that can predict high-risk group in the National Lung Screening Trial dataset, and further validated the model in the TCGA lung adenocarcinoma dataset. More importantly, they showed that these image-derived TME features significantly correlated with the gene expression of biological pathways. For example, transcriptional activation of both the T-cell receptor and programmed cell death protein 1 pathways positively correlated with the density of detected lymphocytes in tumor tissues, while expression of the extracellular matrix organization pathway positively correlated with the density of stromal cells. Taken together, they demonstrated that by applying HSD at cell-level analysis in H&E images, spatial organization of different cell types could be identified and associated with the gene expression of biological pathways.

## An Overview of Generating Weakly Cell-Level Annotation Using IHC Stained Images

### IHC-Based Cell-Level Annotation

Building a dataset using image-level, or region-level annotation is comparably easier than cell-level annotation. Unlike image-level annotation, which is generally reduced to a simple classification task, both region-level and cell-level annotation require the addition of a segmentation step alongside classification. Indeed, the principal difference between region- and cell-level classification is a matter of scale, though this difference is several orders of magnitude in size. Considering that a WSI can easily contain several million cells, completely annotating enough images to train the classifier is almost impossible even for experts. The difficulty in producing cell-level annotations for training data has compelled most groups to use region-level analysis of WSIs to capture cell-level features, such as presence of tumor infiltrating lymphocytes (Saltz et al., 2018). These analyses use region-level annotation and consider that the cells in the annotated region have the same cell types. These approaches can still be informative but considering that tumor is very heterogeneous, and there are multiple types of cells coexists even in a small single region, labeling all the cells in the same region will cause many mislabeled cell-level annotations.

**FIGURE 3:**
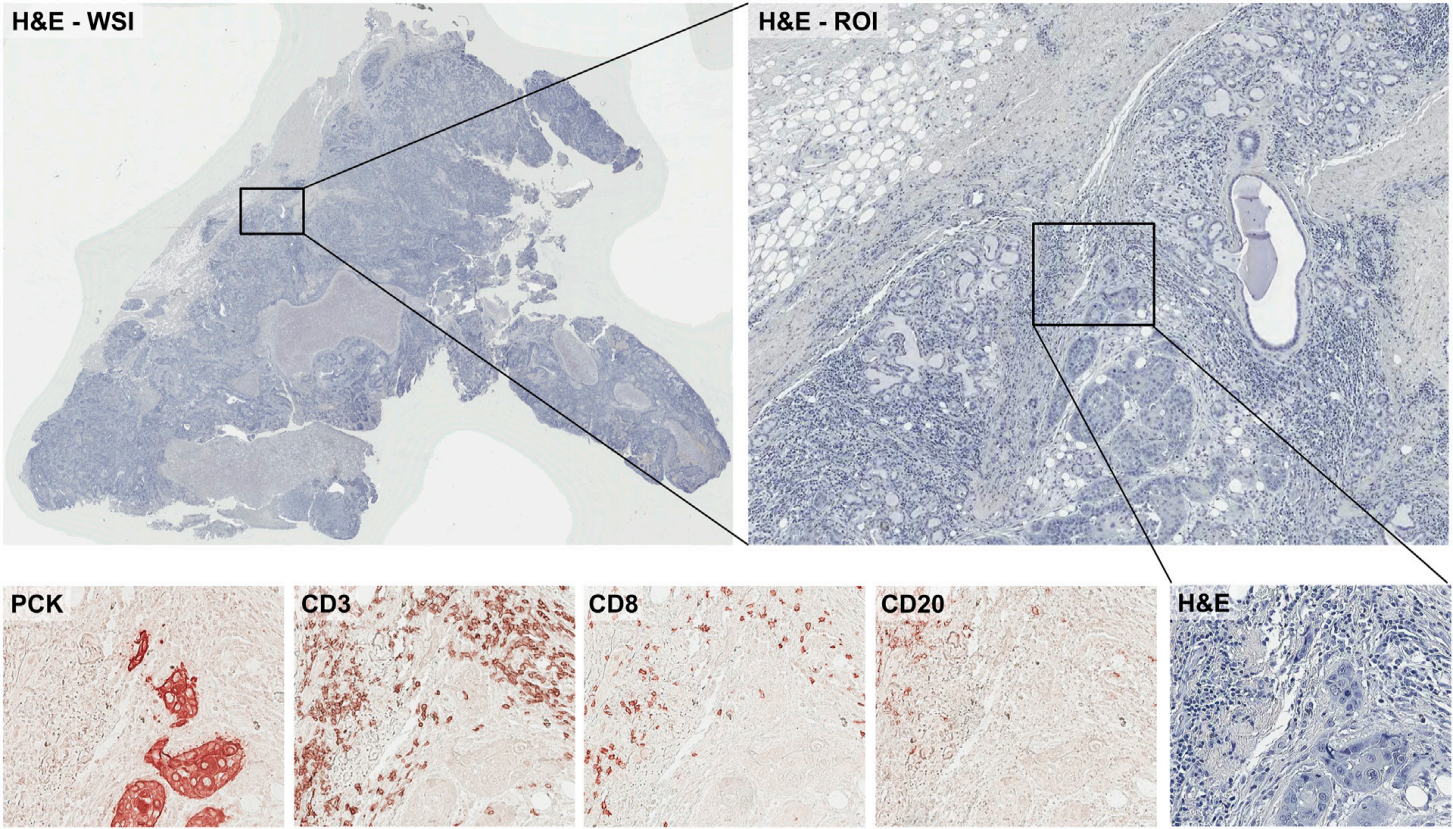
Example of the IHC-stained histopathology images with different antibodies.

As discussed in *Histopathology Image Data Preparation for Training Machine Learning Models* of this review, H&E staining is used frequently for basic examination of tissue and cell morphology. Immunolabeling may also be performed to obtain additional information from samples such as cells’ subtypes. For example, pan-cytokeratin staining (PCK) is commonly used to stain for tumor cells, and some antibodies such as CD3 (T-cell), CD8 (T-cell), and CD20 (B-cell) are used for characterizing immune cells in the sample (Figure 3). Conventional immunohistochemistry (IHC), multiplexed IHC (mIHC), or multiplexed IF (mIF) images can be used for labeling multiple cells in histopathology images. When preparing sections from biopsies or resected tumors, slides will be prepared from a series of adjacent sections. This allows pathologists to easily compare regions of a H&E and adjacent immune-stained slides. Even though the adjacent samples are still showing similar spatial characteristics, they are not identical to the other samples. To perform machine learning data analysis and interpretation, it is critical to align these differently stained histopathology images together. Most conventional image analyses software can perform a reasonable job in aligning these different slices of images and provide a final aligned image to study tumor heterogeneity and tumor immune microenvironment.

To train machine learning classifier for cell-level annotation, images must first be annotated with individual cells’ boundaries and the cells’ subtypes. As explained earlier, cell-level annotation process requires a tremendous manual effort. To make this cell-level annotation more approachable and scalable, we introduce a semi-automated method of generating cell-level annotation using adjacent IHC-stained images as the labeled dataset for machine learning methods. In this example, we are using H&E tumor cell images with multiple IHC stained images to generate a labeled training dataset in Common Objects in Context (COCO) format for Mask R-CNN (He et al., 2017). After training on this automatically labeled training data, the machine learning classifier can predict cell types in H&E image without further IHC or IF stained images with high accuracy. In the following sections we will provide an explanation of the steps required in this approach, beginning with acquisition of the WSIs.

### Digital Image Acquisition From Prepared Slides

Once the histology slides have been prepared using H&E or IHC, they must then be converted to a digital format for analysis by the machine learning model. While such images can be taken using a simple brightfield microscope, the quality and characteristics of the images can vary considerably from one slide to the next. Computer driven microscopes offer a better solution for reproducible image acquisition and can even be used to generate WSIs through stitching of image tiles. However, unless carefully calibrated the resulting WSI may contain significant stitching artifacts that can confuse a machine learning classifier. Instead, it is highly advisable to use a dedicated slide scanner, such as Leica’s Aperio platform, to generate WSIs of stained slides. The H&E and mIHC images used in our example process were all captured on a Leica Aperio AT2 digital whole slide scanner at 20x magnification. This system produces a “.svs” file that contains an image of the slide label, a macro image of the entire slide, and a multi-resolution tiled “.tiff” of the WSI. The structure output files will vary among different slide scanning systems, but most rely on multi-resolution tiled TIFF files to store the WSI. For easier image modification and processing, svs files need to be converted to tiff or png formats.

While the example provided here relies on IHC stained slides, immunofluorescent (IF) labeling can also be used to generate cell-labeled slides. Most of the considerations described above for IHC apply to these approaches, but IF can generally provide a higher number of labels on a single slide. Systems like the Akoya Vectra or the Leica Aperio Versa are capable of scanning whole slides with up to 7-plex labeling. Spectral overlap of the fluorescent reporters presents a unique challenge in multiplexed IF, which was recently reviewed by Shakya et al. (2020). The plexity of both IHC and IF can be increased by sequential staining/de-staining for target proteins. Higher numbers of simultaneous labels (40+) can be achieved by newer techniques such as the Akoya CODEX or multiplexed ion beam imaging (MIBI) that use oligo- or metal-isotope-labeled antibodies, though these techniques require additional components or a specialized secondary ion mass spectroscopy system, respectively. It is also possible to label cells of interest by nucleic acid *in situ* hybridization (ISH). ISH and its fluorescent counterpart (FISH) are generally more laborious than immunolabeling and may not perform well on older samples due to the more sensitive nature of nucleic acids compared to proteins, but image acquisition is performed in the same manner as IHC or IF.

### Image Registration and Normalization

After WSI images are stained with different antibodies are captured, a number of preprocessing steps should be followed to ensure optimal performance of the machine learning classifier, including stain normalization and slide registration. The adherence to a defined protocol for H&E and IHC staining is crucial to minimize the variability between batches of slides, but some level of variability will still occur due to imperfections in tissue sectioning or changes in tissue composition. Staining variability can be compensated for after image acquisition by normalization to a standard using several suggested methods (Macenko et al., 2009; Khan et al., 2014; Bejnordi et al., 2016; Alsubaie et al., 2017; Roy et al., 2018; Anghel et al., 2019). Stain normalization is especially important when incorporating external datasets due to potential differences in staining protocols and image capture systems.

Depending on what sampling/staining method is used, different steps of preprocessing are needed to generate aligned or registered image dataset. Conventional IHC uses adjacent slices of the samples for each staining, and they are not identical. Also, during the sampling, placing, staining, and processing, each section might be moved, rotated, mirrored, or simply imperfectly placed onto the slide which may causes images to be poorly registered (Wang et al., 2014). Since mIHC is stained for the same identical sample for multiple times, the images are comparably more aligned compared to conventional IHC datasets. However, during the staining-washing-placing steps in each repetitive staining, the samples can be slightly displaced or even washed off. Because of that, mIHC images may still not perfectly align and should be registered as well. mIF datasets, in contrast, does not require repetitive washing and staining steps and should therefore be easier to register to adjacent slides.

Image registration is common step in image processing using multiple images especially in biomedical image analysis such as radiology. It is a process of overlaying two or more images from different sources or different time of the same object to align geometrically (Zitová and Flusser 2003). In radiology, this method is used for overlaying images from different sensors, different equipment, or different time (Fox et al., 2008; Tohka and Toga, 2015). This image registration is sometimes done manually using image viewer software when there are not many images, but for multiple image files, automated methods can be used. ImageJ (Rueden et al., 2017) and Fiji (Schindelin et al., 2012) have multiple registration plug-ins such as “Feature Extraction SIFT/MOPS”, or “TrakEM2”. MatLab also has several applications including “Registration Estimator App” and “Intensity-Based Automatic Image Registration”. SimpleITK (Lowekamp et al., 2013) is an open-source image analysis toolkit that supports multiple platform such as Python, R, Java, C#, C++, and Ruby that provides powerful machine learning-based registration options for image registration process. In most of the automated methods, images are aligned globally, which means at a smaller scale parts of the images might be not very well aligned. In this case, additional registration steps can be done after deciding ROIs or split the samples in smaller patches.

### Cell Boundary Detection and IHC Intensity Level Acquisition

For the cell boundary information, many (semi-)automated cell boundary recognition programs can be used. CellProfiler is one of the most well-known histopathology image analysis program (McQuin et al., 2018). CellProfiler supports multiple types of histopathology images as input, and users can build their own pre/post-processing pipeline of the image quantification. Using CellProfiler, cell boundaries can be segmented by setting up some simple parameters such as the typical diameters of the nuclei (or cells), segmentation thresholding methods (e.g., Otsu, Minimum Cross-entropy), smoothing thresholds etc.

No cell segmentation program is perfectly accurate, and depending on the image dataset that are used for this process, the parameters may need to be modified. For example, the cell sizes in the images can vary depending on the zoom level of images, the sampled tissue parts of the organs and species. Finding good parameters to detect the cells with higher accuracy is an important step. For example, the cell smoothing threshold parameter is too high, multiple different cells can be recognized in a single cell. However, if the threshold is too low, a single cell is seldom recognized as multiple cells. These parameters are required to be optimized based on the target image dataset. This might be repetitive and time-consuming part of the image processing, however, finding optimal parameters for each input dataset are important for accurately finding the cell boundaries from the image. When the cell boundaries are detected, CellProfiler outputs the results in a mask image file. This mask image file is showing the detected cells in different colors, and the background as black.

CellProfiler also has the tools to measure staining intensities of each recognized cells from multiple aligned IHC images. For example, after CellProfiler detects cells boundaries in the main input image (e.g., H&E), the staining intensities of the same location are extracted from other IHC images (e.g., PCK, CD14). The intensities in aligned IHC images are obtained in a table with csv format. This table contains the x/y coordinates of the detected cells, and their cell marker intensities obtained from different IHC images. After semi-automated image processing steps, the information of the detected cell boundaries and the IHC intensities of the cells could be saved in mask image files and a csv file, respectively.

### Combine the CellProfiler Information and Decide Cell Class Subtypes

The cell boundary information, and the intensities of each markers in the cells are obtained as two different types of files from the previous step. In this step, the intensity levels need to be converted to cell class labels. Depending on a prior knowledge of domain experts, the rule for cell class labeling can be made. For example, a cell with a high intensity level in PCK stained image will be labeled as a tumor cell. During this labeling rule generation, some markers will require a threshold values to divide positive and negative class cells. Many of the marker values can be shown bimodal distribution in histograms. The local minimum value in between the two modes can be used as the cutoff point for detecting positive/negative staining intensities (usually this cutoff point resides between 0.3 and 0.5). Some of the staining markers may not have bimodal distributions, but have different distributions (e.g., unimodal or multi-modal). In these cases, additional steps may require to establish a biologically-informed decision process based on the advice of domain experts. Following the application of the cell class decision rules, each cell in the csv files will be mapped to a single class label (e.g., tumor cell, immune cell, stromal cell).

### Reformatting the Annotation Dataset Into Common Objects in Context challenge Format

After the cell subtypes are decided, the cell mask image (cell boundary information) and the csv information (cell subtype information) need to be combined and reformatted. The most commonly used annotation file format is called COCO format, which is used in Microsoft’s Common Objects in Context challenge (COCO)^1^ (Lin, Maire et al., 2014). The COCO dataset is one of the most popular object detection datasets with multiple different objects’ images and their annotations. The annotation file format contains image names, object boundary locations, objects’ class name, and the objects’ class label of all the detected objects. Most of the current machine learning-based object detection methods^2,^ ^3,^ ^4^ are using COCO format as the input of the annotation information, and there are multiple publicly available tools for generating, loading and modifying such as COCO API and FiftyOne.

Since the output of CellProfiler is not in COCO format but the mask images, the files cannot be used directly by most of the known image analysis methods. To convert the CellProfiler outputs to COCO format JSON file, the following processing steps are needed. First, the cell mask images must be converted into the list of x/y coordinates that represents the boundary of each cell. FiftyOne which is a Python open-source tool for image dataset building supports the input of mask images and can convert them into coordinates in the image. After the cells’ boundaries are mapped to cell subtype classes, this information needs to be saved as COCO JSON format.

Through this step, the cell mask image and cell class information are converted to a single JSON format that includes cell boundary coordinates, cell classes, and the input image file information.

### Divide Images Into Patches/Train + Test Dataset Generation

Now, the input images with cell-level annotation dataset are ready for use. However, depending on the input image size, the images need to be split into smaller patches or tiles. Most of the deep learning-based image segmentation methods require GPUs with high memory because of the amount of computations in the complex neural network structure. Depending on the available GPU memories of the machine, the size of the input images needs to be modified. Most of the popular deep learning methods take images from 128X128 pixels to 1024X1024 pixels, depending on the parameter settings of the code or GPU memory of the machine. WSI images are several orders of magnitude larger than an individual image patch, and even most of the ROI sections are still bigger than this range. Splitting images is easily accomplished using many of publicly available tools such as Pillow in Python; however, COCO annotation file needs to be re-generated by calculating the new coordinates of the cell boundaries in the split images. Since the cells on the edge of the image are not easily segmented by machine learning methods, it is recommended that the image splitting allows some overlaps between the image patches.

Also, as it is common in machine learning and data science field, before training the dataset with machine learning methods, the dataset needs to be separated into training, testing, and sometimes validating datasets. It is important that the training/testing data separation needs to be done in WSI level, not patch level–which means the patches from the same WSI must not co-exist in both train/test dataset. Adjacent patches from the same WSI can be overlapped, or shares many properties such as shapes, colors, and patterns, which can cause a boosted accuracy scores in the test dataset because the machine learning classifier already have seen very similar (adjacent or overlapped) cells or tissues.

### Training Machine Learning Classifier

Once the dataset is ready, it is time to train a machine learning classifier. There are many machine learning classifiers^5^ ^,^ ^6^ ^,^ ^7^ designed for image segmentation and classification for the COCO challenge, and by changing some parameter settings most of them can be used for this histopathology cell subtype segmentation task.

Deep learning-based machine learning classifiers usually require a large amount of training dataset. If the training dataset is not big enough, a “warm-start” method (pre-training/fine tuning) is highly recommended. For the popular machine learning models, there are pre-trained weights that are trained with big datasets are publicly available. For example, Wang et al. used a pre-trained Mask R-CNN model with COCO dataset and public balloon image dataset, and fine-tuned with their histopathology image dataset for cell segmentation task (Wang et al., 2020). This fine-tuning method (pre-train with big dataset/fine-tune with final dataset) is widely used to overcome the limitation of the lack of training dataset, especially for deep learning model training that requires big training dataset. For Mask R-CNN method, several pretrained weights are publicly available.^8^ ^,^ ^9^

As the goal of the tasks are not the same between balloon detection and cell segmentation, many of the parameters in Mask R-CNN codes needs to be updated to optimize the machine learning classifier to detect cells more accurately before training with the target dataset. For example, in histopathology images there are almost certainly a higher number of the target objects per image (e.g., nuclei or cells) than in other image sets. Therefore, the maximum detection threshold needs to be changed to higher number and the target sizes should be set to smaller, respectively. Also, target objects are taking up much more space in the histopathology images compared to other segmentation tasks, so changing ROI positive ratio will be helpful.

For cell-type detection, class imbalance problem is a known issue in the histopathology analysis. For example, in tumor sample slides, most of the cells are tumors, but only a limited number of cells are immune cells. As class imbalance is a well-known problem in machine learning field, there are several suggestions to solve this problem including over/under sampling, using different cost/weight schema during the training, (Almeida et al., 2014; Johnson and Khoshgoftaar 2019). If an insufficient number of images are included in the training dataset, image augmentation can be employed to synthetically increase the dataset size. Image augmentation generates more images for training dataset by image alteration (e.g., rotation, flip, blur, crop, pad, or adding noise) and is widely used for deep learning methods (Shorten and Khoshgoftaar 2019). In the case of Mask R-CNN, an image augmentation step is built into the pipeline.

In histopathology image method training, there are several things to consider for getting good accuracy of cell segmentation and subtype prediction. Since deep learning methods require very high number of calculation and high memory during training the classifier, it is almost impossible to train classifiers without high performance hardware with GPUs and high memory. Depending on the performance of the hardware and time limitations, the training parameters (such as learning rates, epochs, the layers to be fine-tuned, etc.) require tuning to optimize the performance.

### Evaluation of the Results

After obtaining the prediction results in the test dataset, the results need to be evaluated. To evaluate how accurately the machine learning classifier can find cells’ boundaries and their subtypes, the Intersection over Union (IoU) metric, also known as Jaccard index can be used (Girshick 2015; Ren et al., 2015; He et al., 2017). For each class of subtypes, the intersection of the ground truth area and the predicted area (Rezatofighi et al., 2019). Usually, IoU is calculated based on the bounding box of the segmentation. When IoU is calculated in an image-level, it can be calculated for each class and averaged to see the final image segmentation accuracy for all the classes. This score is called mean-IoU (mIoU). When IoU is used for binary decision of a single object detection, if the IoU is higher than 0.5–0.7, it is considered that the object is correctly detected.

$$IoU=\frac{\#TP}{\#TP+\#FP+\#FN}=\frac{Area\;of\;Ground\;Truth\cap Area\;of\;Predicted}{Area\;of\;Ground\;Truth\cup Area\;of\;Predicted}.$$ (1)

Ultimately, validation and verification of the prediction by a pathologist remains the gold-standard for histopathology image analysis.

### Visualization and Obtaining Biological Insights

**FIGURE 4:**
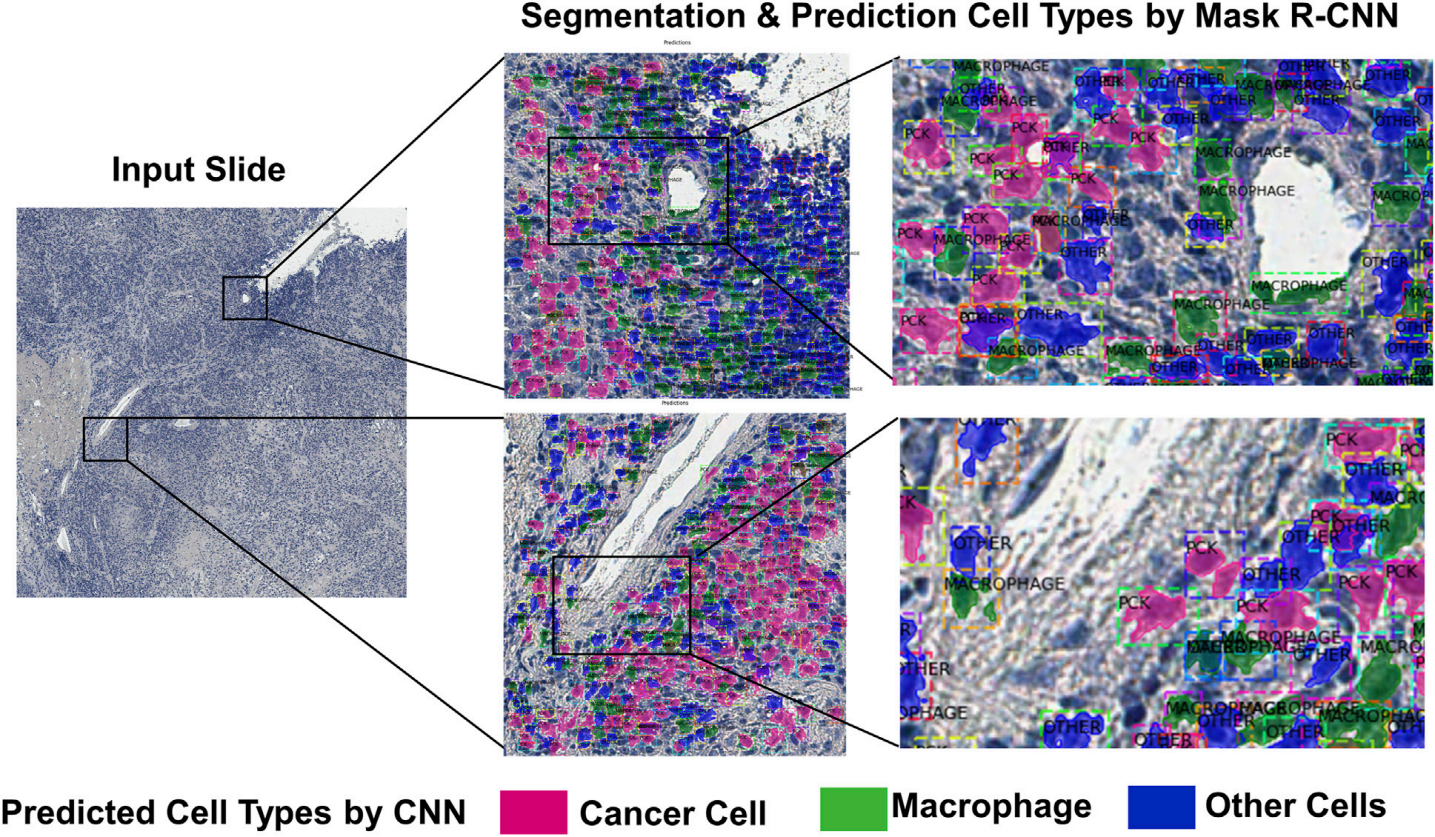
An example of the deep learning-based cell subtype prediction results.

As prediction of the cell subtypes are performed on the image patches, the predictions need to be stitched and rebuilt into the original image. As the classifier predicts the cell boundaries and subtypes, it is possible to overlap the predicted cell segments on the original images with different colors depending on the class. (Figure 4). This can give clinicians the information of tumor heterogeneity or immune infiltration in the sample.

There are several methods to quantify the tumor heterogeneity in the output image. Ripley’s K function (Ripley 1988) that is a function of calculating spatial point pattern by finding number of other points within a circle of radius, is used for several histopathology image analysis (Mattfeldt et al., 2009; Yuan et al., 2012; Carstens et al., 2017). The Shannon diversity index (Shannon 1948) is also widely used for calculating the diversity of molecules or distribution of species. Graf and Zavodszky suggested a method for calculating molecular entropy and heterogeneity diversity metrics based on the Shannon diversity index for quantifying tumor heterogeneity (Graf and Zavodszky 2017). These methods can be calculated at the patch-level or for image-level analysis using sliding window.

### Pros and Cons of IHC-Based Weakly Cell-Level Annotation

Using registered H&E and IHC slides can provide a good alternative to cell-level manual annotations. This technique allows for easy production of a large dataset of “weakly labeled” images with a much better annotation resolution than single class region annotation for cell-level machine learning classifiers. Indeed, for applications with rare or non-contiguous organization, such as identification of immune cell types within tumours, region-level annotations are unlikely to produce a usable training set. However, cell-level registration has its own limitations on the accuracy of the resulting labels. In the simplest case, cell-level registration with a single marker on each slide from typical IHC, it may be necessary to register multiple slides of various distances to a single H&E image. It stands to reason that the greater the distance between the two registered slides, the less accurate the registration will be. Multiplexed or sequential immunolabeling, such as the mIHC approach used in our example, can mitigate this loss of accuracy. If only a few labels are needed to construct the training dataset it would be possible to generate a nearly perfect cell-level registration by digitizing the H&E slide, de-staining, and then re-staining using IHC (Hinton et al., 2019).

There are several other difficulties to consider while designing the machine learning models. Cell-level histopathology analysis can be prone to cell class imbalance problem, because some of the specific types of cells might be very rare compared to the other tumor or normal cells. There are several methods to handle class imbalance in machine learning (Johnson and Khoshgoftaar 2019), however, manually checking the distribution of the class labels are recommended. Manual validation process is also recommended to remove some low-quality images or images with noises. For example, sometimes during the staining or washing step of the sample preparation, some samples are missing, or air bubble can be formed in the image. These noises can cause bias in the training steps, and manual validation of images is recommended to remove these low-quality samples.

## Future Direction of the Histopathology Image Analysis at Single-Cell Level With Deep Learning

As shown in several publications, deep learning-based histopathology analysis is becoming a useful tool for tumor image data analysis. Most of the publication are focusing on classification of the sample in image, region or cell level, however, this deep learning-based histopathology techniques can be expanded into more broad topics of research.

Machine learning-based image analysis can be used to predict transcriptomics information from the image. Schmauch et al. (2020) demonstrated a machine learning model trained with bulk RNAseq data to predict spatial gene expression from H&E WSI images. With the recent development of spatial transcriptomics techniques such as LCM-seq or 10X Visium Spatial Gene Expression, it is possible to obtain the gene expression of the small region in the sample with the histopathology image together. These spatial transcriptomics data with paired histopathology images can be a great training dataset for gene expression prediction from histopathology image of the sample. With this dataset of spatial gene expression and images with deep learning methods make it available to predict gene expression of the specific region from its histopathology image. He et al. (2020) used spatial genomics dataset of 23 breast cancer patients to predict gene expression from the histopathology of small regions. The results are promising though limited because of the small size of the dataset. After collecting more dataset from these spatial genomics datasets with images, the gene expression prediction will certainly be improved. The scope of these prediction will be eventually combined with single cell RNA-seq dataset for cell-level prediction. Currently, the technical and cost limitation of single cell technology, it is almost impossible to generate enough dataset for cell-level annotations for deep learning dataset, however, in the future after the limitation is removed, cell-level expression prediction will be possible.

As histopathology images include a lot of information, and it can be used for predicting further information. Histopathology images may include rich information such as tumor grade, tumor subtype, immune infiltration, and so on. This information may give clinicians some idea for finding the personalized treatment for each patient. As an example, Wang et al. (2020) predicted overall survival of the patients of the samples using deep learning-method.

As explained earlier, small training dataset problem can be mitigated by training machine learning models with a big dataset that is similar to the target dataset followed by fine tuning with the target dataset. For this purpose of pretraining deep learning methods, public dataset of histopathology images will be very helpful for researchers who are having lack of training dataset issues. Komura et al. introduced several WSI datasets that are publicly available (Komura and Ishikawa 2018), and The Cancer Image Archive (TCIA) is also a good place to find the similar image datasets to pretrain the model. One major problem of these publicly available image dataset is that the images are generated and processed in many ways, which makes it harder for combining them to construct a big training dataset. Some standardized steps of generating and pre-processing image datasets will make the public image dataset more valuable and easier to use for machine learning model training.

In conclusion, building a machine learning model for histology image analysis requires a significant investment of time and effort on both the computational and the biological side. A basic understanding of the biological and data science principles that underpin these methods is key to establishing a productive multi-disciplinary team of researchers for this promising and rapidly growing field. In addition, as the amount of WSIs and associated molecular data becoming widely available to researchers, the development and application of computational approaches will become more robust and reproducible. We are optimistic that these computational approaches will play an important role to uncover the insights contained in these histopathology image datasets.

## Footnotes

1
https://cocodataset.org

2
https://github.com/matterport/Mask_RCNN

3
https://github.com/facebookresearch/Detectron

4
https://github.com/endernewton/tf-faster-rcnn

5
https://github.com/matterport/Mask_RCNN

6
https://github.com/facebookresearch/Detectron

7
https://github.com/endernewton/tf-faster-rcnn

8
https://github.com/matterport/Mask_RCNN/releases/download/v2.0/mask_rcnn_coco.h5

9
https://github.com/matterport/Mask_RCNN/releases/download/v2.1/mask_rcnn_balloon.h5
